# Comparison of supine or prone crawl photon or proton breast and regional lymph node radiation therapy including the internal mammary chain

**DOI:** 10.1038/s41598-019-41283-1

**Published:** 2019-03-18

**Authors:** Bruno A. Speleers, Francesca M. Belosi, Werner R. De Gersem, Pieter R. Deseyne, Leen M. Paelinck, Alessandra Bolsi, Antony J. Lomax, Bert G. Boute, Annick E. Van Greveling, Christel M. Monten, Joris J. Van de Velde, Tom H. Vercauteren, Liv Veldeman, Damien C. Weber, Wilfried C. De Neve

**Affiliations:** 1 Department of Radiotherapy and Experimental Cancer Research, Faculty of Medicine and Health Sciences, Ghent University, Ghent, Belgium; 2 Paul Scherrer Institut, Villigen, Switzerland; 3 Department of Radiation Oncology, Ghent University Hospital, Ghent, Belgium; 4 Department of Anatomy, Faculty of Medicine and Health Sciences, Ghent University, Ghent, Belgium; 5 Industrial Design Center, Faculty of Engineering and Architecture, Ghent University, Ghent, Belgium; 6 Radiation Oncology Department, University Hospital of Bern, Bern, Switzerland

## Abstract

We report on a dosimetrical study comparing supine (S) and prone-crawl (P) position for radiotherapy of whole breast (WB) and loco-regional lymph node regions, including the internal mammary chain (LN_IM). Six left sided breast cancer patients were CT-simulated in S and P positions and four patients only in P position. Treatment plans were made using non-coplanar volumetric modulated arc photon therapy (VMAT) or pencil beam scanning intensity modulated proton therapy (IMPT). Dose prescription was 15*2.67 Gy(GyRBE). The average mean heart doses for S or P VMAT were 5.6 or 4.3 Gy, respectively (p = 0.16) and 1.02 or 1.08 GyRBE, respectively for IMPT (p = 0.8; p < 0.001 for IMPT versus VMAT). The average mean lung doses for S or P VMAT were 5.91 or 2.90 Gy, respectively (p = 0.002) and 1.56 or 1.09 GyRBE, respectively for IMPT (p = 0.016). In high-risk patients, average (range) thirty-year mortality rates from radiotherapy-related cardiac injury and lung cancer were estimated at 6.8(5.4–9.4)% or 3.8(2.8–5.1)% for S or P VMAT (p < 0.001), respectively, and 1.6(1.1–2.0)% or 1.2(0.8–1.6)% for S or P IMPT (p = 0.25), respectively. Radiation-related mortality risk could outweigh the ~8% disease-specific survival benefit of WB + LN_IM radiotherapy for S VMAT but not P VMAT. IMPT carries the lowest radiation-related mortality risks.

## Introduction

Radiotherapy after breast-conserving surgery improves loco-regional control and survival at the expense of acute and late local toxicity, radiation induced cardiac injury, lung cancer and cancer in the non-treated breast, leading to dose-dependent excess mortality^1–6^. Prone radiotherapy allows decreasing dose to lung and heart and thereby the risks of radiation-induced lung cancer and cardiac injury^7–9^. Prone breast radiotherapy devices are typically designed to support the patient with both arms elevated. The elevated arm at the treated side and device components that support the arm restrict the range of beam directions for whole breast and lymph node irradiation (WBI + LNI). Prone WBI + LNI using dorsal beams has been described^10^. Heart and lung dose from WBI + LNI are highest if the internal mammary(IM)-lymphnodes are also irradiated^11,12^. Long-term mortality risk from radiation-induced lung cancer and heart injury increases linearly with mean dose to these organs^13^. The concern that possible benefits from IM-irradiation^14^ might be offset by radiation-induced heart injury explains reluctance of radiation oncologists to treat the IM-nodes.

We developed a support device for a particular prone position with the patient’s arm alongside the body at the treated side and above the head at the contralateral side^15^, resembling a phase of prone crawl swimming. The device, called prone crawl breast couch, allows WBI + LNI using anterior, antero-lateral and para-sagittal beam directions without the patient’s arm or device components in the entrance paths of the beams^15^. We showed that prone crawl WBI + LNI (without inclusion of the IM-chain in the lymph node target) reduced heart and lung doses as compared to supine techniques^16^. The purpose of this study was to explore the potential of the prone crawl position for organ-at-risk (OAR) sparing in left-sided WBI + LNI (including the IM-chain) using photon or proton irradiation.

## Patients, Materials and Methods

Ten left sided breast cancer patients with invasive carcinoma of the breast and pathologically confirmed positive lymph node status were included in this retrospective study. Patients were not selected for large or pendulous breasts. All patients underwent lumpectomy and axillary clearance followed by adjuvant WBI + LNI in supine position. Written informed consent was obtained before simulation for each patient. The study was conducted according to the Declaration of Helsinki and was approved by the ethics board of Ghent University Hospital. Six patients were scanned in supine and prone crawl position and 4 patients only in prone crawl position. In prone crawl position, patients were positioned on the prone crawl breast couch^15,16^. The contralateral breast was pulled laterally, away from the ipsilateral breast and immobilized using a unilateral bra to increase distance to the irradiated area^7^. In supine position, patients were positioned on the simulator couch using a Posirest arm support (Civco Medical Solutions, Kalone, Iowa, United States) with both arms elevated above the head^16^. For both positions, 5 mm slice thickness CT-images were acquired, starting at the vertex and caudally ending below the diaphragm.

The target consisted of the breast, the level II-III axillary (AxII-III), inter-pectoral, peri-clavicular (PC) and IM lymphnode regions. Delineation was performed on a Pinnacle 9.8 treatment planning system (Philips Healthcare, Fitchburg, Wisconsin, United States). In supine position, the contouring guidelines from the ESTRO and PROCAB groups^17–19^ were used to define a clinical target volume for whole breast irradiation (CTV_WBI) and two clinical target volumes for lymph node irradiation. The whole breast was delineated up to 5 mm from the skin surface as CTV_WBI. CTV_PC included axillary level II-III, inter-pectoral and peri-clavicular lymph nodes. CTV_MI included the IM lymphnodes. As there are no generally accepted guidelines for delineation in prone position, we performed delineation of CTV_WBI, CTV_PC and CTV_MI using extrapolation from the guidelines described above.

Planning target volumes PTV_PC and PTV_MI were obtained as 3 mm and 1 mm isotropic expansion of CTV_PC and CTV_MI, respectively. PTV_WBI was created using a 5 mm margin except in the medial direction to avoid irradiation of the contralateral breast and towards the skin surface to minimize build-up effects. Photon plan optimization structures were created to reduce influence of dose buildup underneath the skin on plan optimization and to secure flash.

The heart was delineated according to Feng *et al*.^20^. The left anterior descending artery (LAD) and the apex were individually delineated. Left and right lungs were contoured separately using the Hounsfield Units options provided in Pinnacle³ 9.8 with threshold 800–4096. Contralateral breast was delineated up to the skin. Esophagus was delineated, starting cranially from the inferior margin of the cricoid and ending inferiorly at the gastro-esophageal margin.

Photon plans used 6 MV X-ray beams which deposit little dose at the skin surface. In the first millimeters below the skin surface, the dose deposition rapidly increases. This characteristic, called build-up permits to deliver high photon doses at depths more than ~5 mm below the skin without severe skin toxicity. Proton beams do not show build-up but techniques like pencil beam scanning can be employed to decrease skin dose. Dose was calculated in stacked 1 mm thick layers up to a depth of 5 mm for photon and proton plans. These layers were created by the following procedure. The intersection of a 2 cm isotropic expansion of CTV_WBI with a 5 mm thick slice immediately below the skin surface was called OAR-skin. The parts of OAR-skin extending 0–1, 1–2, 2–3, 3–4 and 4–5 mm inside the skin surface defined 5 layers called OAR-skin-d1-d2 where d1 and d2 are depths of the superficial and deep surfaces for each of the different layers, respectively. Mean dose in each layer is reported.

Photon plans were created and optimized using planning tools integrated into the GRATIS™ treatment planning platform (Sherouse systems, Inc., Chapel Hill, NC, USA)^21^. GRATIS™ and its history is described in the curriculum vitae of George Winthrop Sherouse [http://gwsherouse.com/GWS/cv.pdf]. The tools for VMAT-planning that were developed at Ghent University as extensions of the GRATIS™ planning platform are described elsewhere^22^. Plans were made using a supine coplanar or prone non-coplanar multiple overlying partial arc VMAT technique to exploit good beam directions and reduce low-dose spread to the organs at risk^15,16^. The final dose calculation was performed using the convolution-superposition dose computation engine in Pinnacle 9.8.

The proton plans were computed on the PSIPlan Treatment Planning System (TPS) for a proton Pencil Beam Scanning (PBS) gantry which is a PBS treatment unit with an upstream energy selection design and fast, double parallel scanning, with beam widths between 2.5 and 4.5 mm σ in air across the 70–230 MeV available energy^23^. An electronically controlled range-shifter of 4 cm water-equivalent thickness, mounted in the nozzle, can be remotely positioned in the beam path, allowing the delivery of Bragg peaks close to the patient surface. The range-shifter can be set either ‘in’ or ‘out’ for the delivery of all spots within a single field, or it can be set in ‘automatic mode’, being introduced per single spot when required, i.e. only when the spots need to be located at depths <4.3 cm, not reachable with energy >70 MeV.

The proton pencil beams (PBs) are initially placed inside the patient on a rectilinear grid. Then only a sub-selection of them covering the target with an isotropic expansion of 5 mm are optimized. Intensity Modulated Proton Therapy (IMPT) was used, with simultaneous optimization of all the spots for all the fields^24,24^. The resulting dose distribution is homogenous within the target, but this is obtained by the combination of single field non-uniform dose distributions.

For prone cases 3 oblique fields coming from below the treatment couch were used with about 30° of angular spread between them. For the supine cases, one direct anterior and two narrow oblique-lateral fields with about 20° to 25° of angular spread were used. No couch rotation was applied for any of the cases. The range-shifter was set in ‘automatic’ mode for all fields and the drift-space between the exit of the nozzle and the patient’s surface was minimized as much as possible so that the beam width of the superficial PBs couldn’t be further deteriorated by the multiple coulomb scattering already occurring in the thick range-shifter material.

In order to avoid having to generate individual plans for each of the treated regions, an ad-hoc structure, which was a union of individual target volumes, was created in PSIPlan. Each field was calculated on that structure and finally the weights of the PBs of all fields composing the plan were optimized together so as to achieve the most homogeneous dose distribution within it, but no proton PBs were placed in between the individual PTVs when they were sufficiently separated (>5 mm) from each other, reducing the amount of healthy tissue irradiation. This technique is called ‘Combined Target’ technique. No dose constraints to OARs were needed for the dose optimization, except for 2 prone patients for which constraints on the skin, thyroid and esophagus were used. The used PBs grid for dose calculation was of 4 mm across the plane transversal to the beam direction and 2.5 mm along the beam axis. The final optimized plans were normalized prescribing 100% of the dose to the combined PTV.

A median dose of 40.05 Gy(GyRBE) in 15 fractions was prescribed to the optimization structures related to PTV_WBI, PTV_PC and PTV_MI. The objective was to have 95% of the volumes covered with at least 95% of the prescribed dose (i.e. 38 Gy) and no more than 5% receiving 105% of the prescribed dose (i.e. 42 Gy).

Dose statistics are referred to as D*n* (the minimal dose delivered to *n* % of the volume) or V*n* (the volume percentage receiving ≥*n* Gy). D2 and D98 were used as surrogates for maximum and minimum dose, respectively.

Thirty-year mortality risk from radiation-induced cardiac injury and lung cancer was calculated according to Taylor *et al*.^13^. Cumulative risk of dying from heart disease and/or lung cancer was calculated as 1-(1-P_h_)(1-P_l_) where P_h_ and P_l_ are the risks to die from radiation induced heart disease or lung cancer, respectively.

## Results

Average breast volume (CTV_WBI) in prone and supine position was 462 (10 patients: range 181–738) cc and 496 cc (6 patients: range 213–733) cc, respectively.

**Figure 1:**
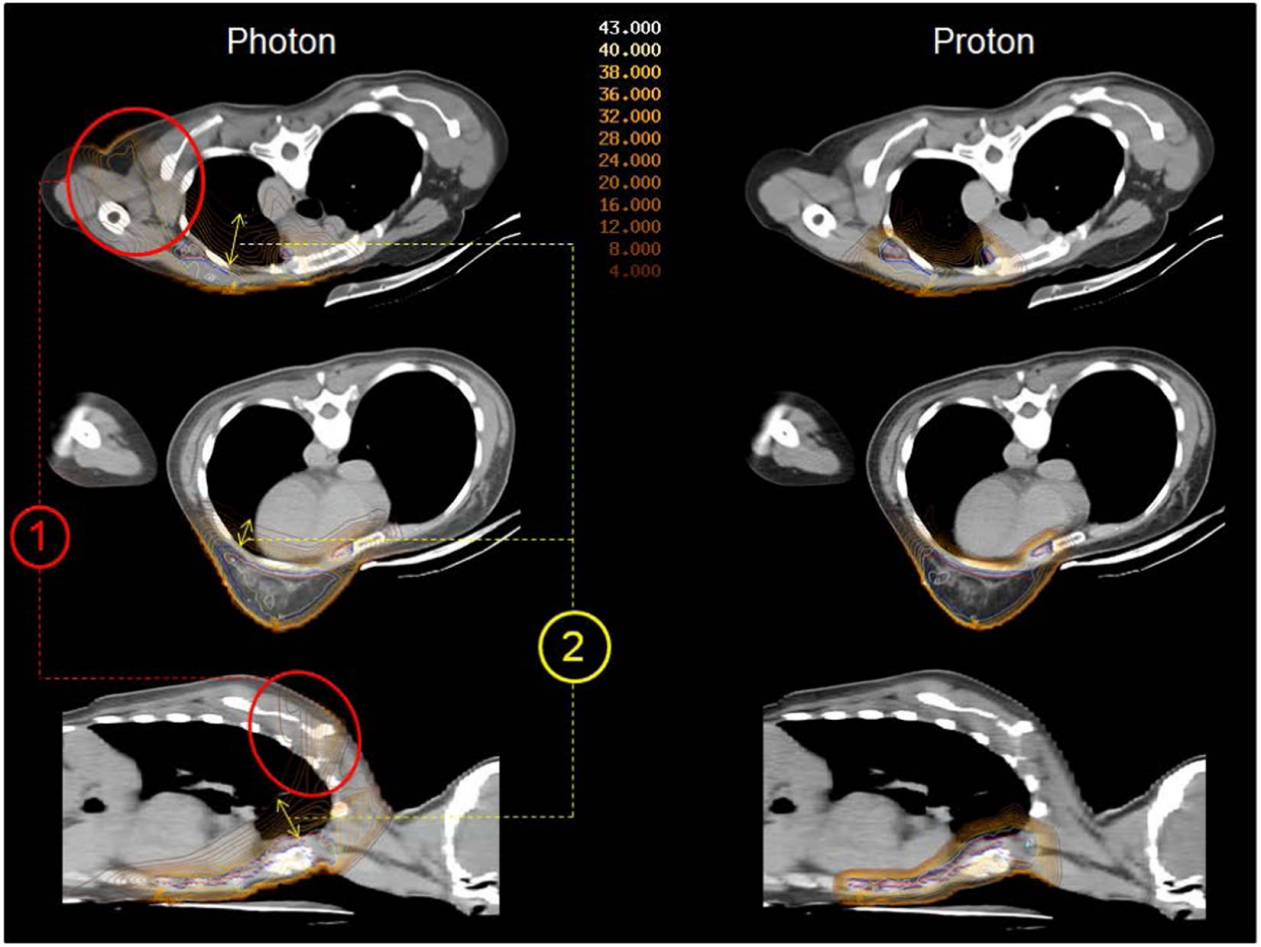
Transverse and sagittal dose distributions of prone photon (left) and proton (right) plans. The main difference is larger dose spread outside the target volumes in VMAT than in IMPT plans. Larger dose spread is seen in de dorsal shoulder region (indicated by the red number (1) and inside the thorax (indicated by the yellow number (2).

Figure 1 illustrates differences between prone crawl VMAT and IMPT. These are build-up, dose spread in the shoulder and intrathoracic regions at the treated side. Similar dosimetric differences between VMAT and IMPT are observed in supine position (not shown).

**Figure 2:**
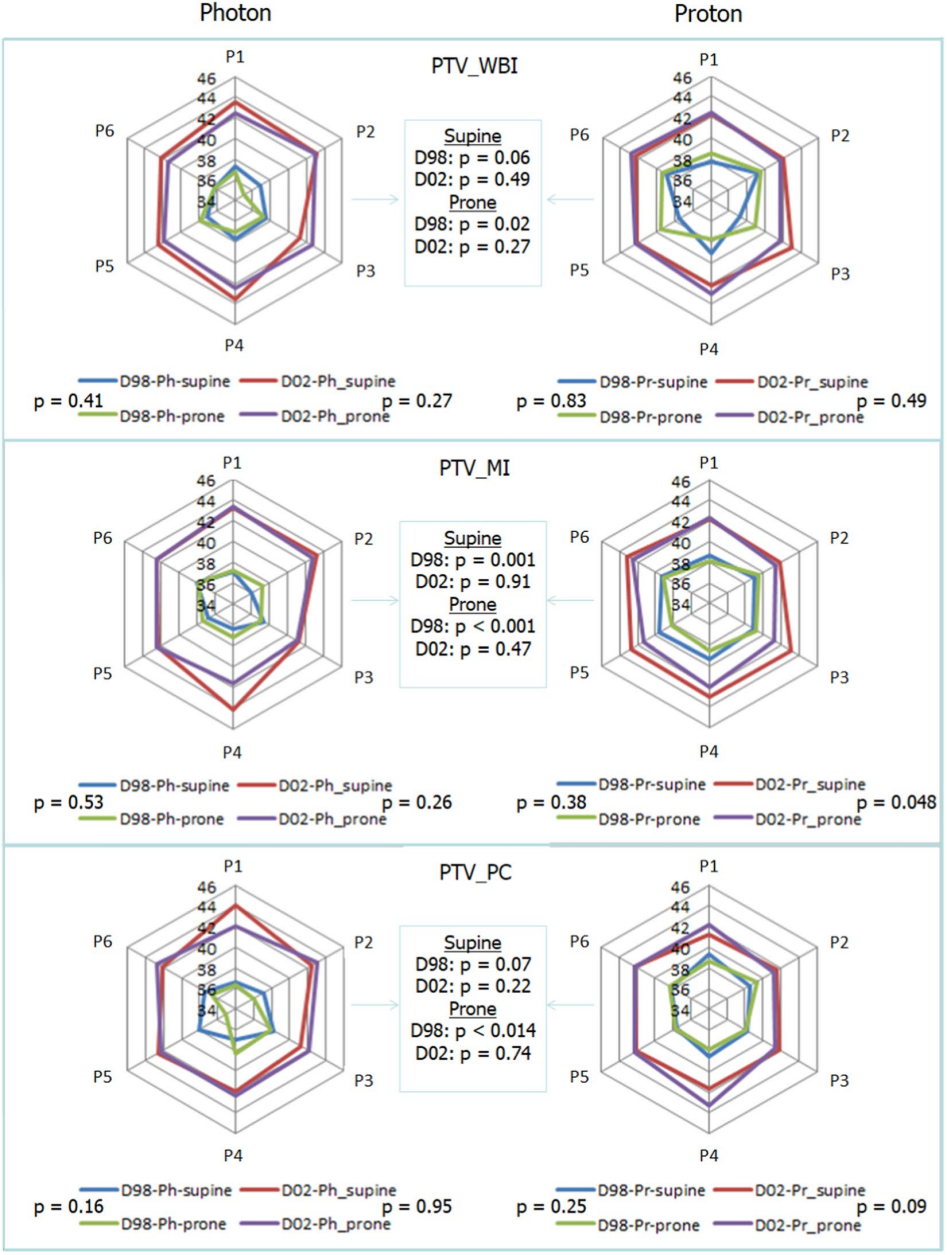
Maximum (D02) and minimum (D98) dose in breast and lymph node targets. Dose range: 34–46 Gy/GyRBE. P1–P6: patients (n = 6) for whom CT-simulation was performed in supine and prone crawl positions. Paired t-tests.

Dose objectives were met for PTV_WBI, PTV_PC and PTV_MI in all plans. Figure 2 shows no significant differences between photon and proton plans regarding the maximum PTV_WBI and PTV_PC doses. For PTV_MI, the average dose maximum was about 1 GyRBE higher in supine than in prone crawl proton plans (p = 0.048). The minimum dose to the target volumes was higher in proton plans than in photon plans. Comparable maximum and higher minimum target doses resulted in better dose-homogeneity for proton than for photon plans in supine as well as in prone crawl position.

Dose indices of OARs are summarized in Table 1. Mean doses to OARs are lower for prone crawl than for supine positions and for proton than for photon plans. The few exemptions are thyroid and esophagus. The lowest average mean thyroid dose was obtained in prone crawl photon plans (statistically significant versus all other plans). The lowest average mean and D02 esophageal dose was obtained in supine proton and supine photon plans, respectively.

**Table 1:** Dose indices for organs-at-risk.

Dose (Gy(RBE))	Photon			Proton			Ph/Pr sup	Ph/Pr pro
	supine	prone crawl	p-value	supine	prone crawl	p-value	p-value	p-value
Heart (mean)	5.6 (3.5–8.8)	4.3 (3.0–5.6)	0.16	1.02 (0.6–1.6)	1.08 (0.6–1.9)	0.8	<0.001	<0.001
LAD (mean)	21.4 (14.8–26.7)	9.0 (4.1–15.8)	<0.001	3.4 (0.7–11.6)	2.0 (0.02–10.9)	<0.001	<0.001	<0.001
Heart apex (mean)	15.6 (10.2–29.4)	13.3 (4.1–25.3)	0.54	4.3 (0.11–13.0)	5.3 (0.04–16.4)	0.72	0.002	<0.001
Lungs (mean)	5.91 (4.1–7.8)	2.90 (2.1–3.9)	0.002	1.56 (1.1–2.0)	1.09 (0.7–1.7)	0.016	<0.001	<0.001
Lung ipsilateral (mean)	11.54 (9.1–15.7)	5.3 (4.0–6.5)	<0.001	3.5 (2.5–4.3)	2.4 (1.6–3.4)	<0.001	<0.001	0.007
Lung contralateral (mean)	1.6 (0.73–5.4)	0.91 (0.39–1.8)	0.4	0.12 (0.02–0.25)	0.04 (0.02–0.06)	0.15	0.1	<0.001
Thyroid (mean)	11.61 (4.1–21.3)	3.31 (0.8–6.2)	0.019	7.18 (2.8–10.5)	6.32 (1.1–11.0)	0.6	0.04	0.01
Esophagus (mean)	2.8 (1.8–6.3)	2.5 (1.1–4.6)	0.71	1.4 (0.2–2.8)	2.2 (0.6–5.1)	0.21	0.11	0.5
Esophagus (D02)	11.8 (3.6–26.4)	14.9 (2.8–34.5)	0.52	14.7 (3.2–27.7)	20.5 (7.2–35.7)	0.23	0.43	0.005
Esophagus (D02<20 Gy(RBE))	5/6 patients	9/10 patients		4/6 patients	5/10 patients			
R breast (mean)	2.0 (0.8–4.6)	0.7 (0.2–1.1)	0.07	0.03 (0.02–0.05)	0.04 (0.02–0.07)	0.15	0.01	<0.001
R breast (mean < 1 Gy(RBE))	1/6 patients	8/10 patients		6/6 patients	10/10 patients			

Average values (range) for 6 or 10 patients. Column 4: p-values for photon supine versus photon prone crawl plans (unpaired t-test). Column 7: p-values for proton supine versus proton prone crawl plans (unpaired t-test). Column 8: p-values for supine versus prone crawl photon plans (paired t-test). Column 9: p-values for supine versus prone crawl proton plans (paired t-test).

**Figure 3:**
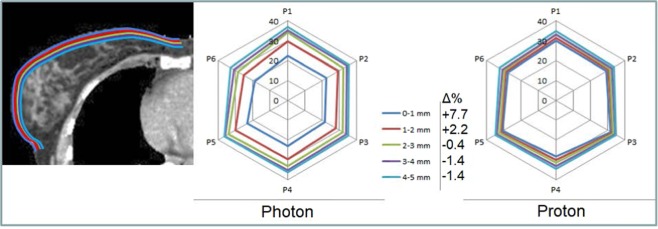
Skin dose. Dose range: 0–40 Gy/GyRBE for patients 1–6. Color lines drawn on the CT-scan image represent the different layers OAR-skin-0–1mm to OAR-skin-4–5mm. Data points are mean dose to these structures.

Mean dose to the different layers of OAR-skin is shown in Fig. 3. The average mean dose for the most superficial layer, OAR-skin-0-1 mm, is 7.7% lower for photon than for proton plans (p < 0.001). The values of OAR-skin-0-1 mm underestimate the sparing by photon build-up of the epithelium which is less than 0.2 mm thick at the breast. For OAR-skin-1-2mm, the difference is 2.2% (p = 0.018). The build-up of photons cannot be distinguished for layers deeper than 2 mm.

Thirty-year risk estimates of dying from radiation induced heart disease or lung cancer for a 50-year old reference patient are given in Table 2.

**Table 2:** Risk estimations for radiation-induced mortality.

WBI + LNI + MI	Photon VMAT		Proton IMPT	
	Supine	Prone-crawl	Supine	Prone-crawl
Heart_mean dose (Gy(RBE))	5.6	4.34	1.02	1.08
**No cardiac risk factor-no smoking**				
ΔRisk cardiac death (0.075%/Gy(RBE))	0.42	0.3255	0.0765	0.081
ΔRisk cardiac death (1/N)	238	307	1307	1234
**Cardiac risk factor(s) or smoking**				
ΔRisk cardiac death (0.3%/Gy(RBE))	1.68	1.302	0.306	0.324
ΔRisk cardiac death (1/N)	59	76	326	308
Lungs_mean dose (Gy(RBE))	5.91	2.9	1.56	1.09
**No smoking**				
ΔRisk lung cancer death (0.06%/Gy(RBE))	0.3546	0.174	0.0936	0.0654
ΔRisk lung cancer death (1/N)	282	574	1068	1529
**Continuing smoking**				
ΔRisk lung cancer death (0.88%/Gy(RBE))	5.2008	2.552	1.3728	0.9592
ΔRisk lung cancer death (1/N)	19	39	72	104
Heart disease*lung cancer mortality				
In high-risk patients: 1 - ∏(1-p)(%)	6.8	3.8	1.6	1.2
	~8% benefit			

Risk estimations for radiation-induced mortality, based on Taylor *et al*.^13^. Over a 30-year period for a 50-year old (reference) patient, the absolute risk of radiation-induced cardiac mortality was estimated 0.075%/Gy and 0.3%/Gy mean heart dose for patients without and with cardiac risk factors, respectively. For radiation-induced lung cancer mortality, the risk was estimated 0.06%/Gy and 0.88%/Gy mean lung dose (both lungs) for patients who never smoked or continued smoking since adolescence, respectively. These rates, multiplied with the average mean heart or lung dose in Gy or GyRBE give an idea of the radiation-induced cardiac or lung cancer mortality risk, respectively, for the different groups. The rows showing Δrisk cardiac or lung cancer death (1/N) give the values of N where 1 out of N reference patients treated would die from radiation-induced cardiac injury or lung cancer, respectively, during a 30-year follow-up period. The heart disease*lung cancer mortality in high risk patients is the cumulative 30-year risk in patients who have cardiac risk factors and continue smoking. Mortality risks are compared to the disease-specific survival benefit of adjuvant WBI + LNI including IM, which we assumed to be ≥8% at 30 years.

## Discussion

The absolute 15-year disease-specific mortality reduction by adjuvant radiotherapy was estimated to be 3.8% in patients receiving radiotherapy after breast-conserving surgery, the majority being node-negative^26^. Absolute 20-year disease-specific mortality was reduced ~8% after mastectomy and radiotherapy in node-positive patients^27^. Non-randomized evidence exists that disease-specific mortality reduction could be equal^28^ or even larger^14,29^ if the IM-nodes are also treated. After adjuvant radiotherapy, the lower overall than disease-specific survival rates can be partially explained by mortality from radiation-induced cardiac disease and secondary cancer, mainly lung cancer. In patient groups with risk factors for developing cardiac disease or lung cancer, adjuvant radiotherapy may even be detrimental^13^. The excess mortality risk increases with mean dose to heart and lungs with no threshold^5,13^. These insights are the rationale behind research exploring techniques to decrease dose to OARs and cast doubt on the use of planning OAR-constraints below which no further effort would be needed. Techniques include prone positioning, deep-inspiration breath hold (DIBH), non-coplanar beam directions, short-arc VMAT, narrow PTV-margins in combination with IGRT and proton therapy. Prone crawl position offers the possibility to use several techniques simultaneously in patients requiring breast and lymph node irradiation. This study focused on breast and lymph node irradiation including the IM-nodes because these patients receive the highest OAR-doses^11^ and because recent publications may lead to more IM irradiation^14,29^. The novelties investigated in this study are i) use of non-coplanar VMAT using arc directions that were made possible by a prone crawl breast couch^15^ (ii) comparison between supine and prone position for photon WBI + LNI (with IM-nodes); (iii) comparison between photon and proton radiotherapy for both patient set-ups.

Coverage of the breast and nodal targets was equally achieved in supine and in prone crawl position. However, the minimum dose in proton plans was higher for all targets, leading to better dose homogeneity than in photon plans in supine as well as in prone crawl positions. The lower minimum dose in photon plans cannot be explained by photon build-up as this affects only the most superficial 2 mm whereas dose is reported for target volumes after exclusion of the most superficial 5 mm.

Dose indices that have been related to cardiac injury and mortality (mean and maximum doses to the heart, LAD and apex) were generally lower for prone crawl than for supine positions and lower for proton than for photon plans. A drawback of this study is the absence of CT-simulation images in DIBH for supine and/or prone positions. In the period 2003–2013, mean heart doses from loco-regional irradiation including the IM-chain, were estimated to be around 2.5 GyRBE using protons and around 8 Gy or 4 Gy using photons without or with breathing control, respectively^11^. Mean heart dose in prone crawl position without breathing control (4.3 Gy (3.0–5.6)) is similar to the values reported with breathing control^11^ and is about half of the 8.7 Gy mean heart dose reported for left-side prone tomotherapy plans by Kainz^30^. Using DIBH in prone crawl position we achieved mean heart doses below 2.5 Gy for all patients who were treated at the left IM-chain using photons (unpublished data). However, 8–12 DIBHs of 15–25 seconds were required to complete a non-coplanar VMAT treatment. Not all patients are capable of such repeated DIBH maneuvers. Prolonged DIBH or jet-ventilation techniques^31,32^ are evaluated. In proton plans, mean heart doses were less than 2 GyRBE in all patients. As compared to photons, proton plans showed 3–5-fold reduction of mean heart, LAD and apex doses.

Risk estimates for radiation-induced cardiac mortality (Table 2) show rates <0.5–0.1% in patients without cardiac risk factors, which can be considered low when compared to the disease-specific survival gain of ~8% for loco-regional adjuvant radiotherapy. In patients with cardiac risk factors, radiation-related cardiac mortality would affect >1% of patients treated with photons and many more would be at risk of suffering from radiation-related non-fatal cardiac events during follow-up. According to Smolina *et al*.^33^, the death risk rate (myocardial infarction/death from myocardial infarction) is around 5 in women <75 years of age (case-fatality rate = 0.19 in 2010). Using a death risk rate of 5, more than 6% of high-risk patients would suffer from major cardiac event(s) during 30-year follow-up after photon treatment.

The main advantage of prone radiotherapy is lowering lung dose. Risk estimations for radiation-induced lung cancer mortality (Table 2) show rates <0.4–0.1% in patients who did never smoke. In a patient 50 years old at diagnosis, who smoked since adolescence and continued smoking, 30-year radiation-related lung cancer mortality would be more than 5% when treated with photons in supine position and around 2.5% when treated in prone crawl position. Proton therapy reduces lung cancer mortality risk to less than 1.5% and 1% in supine or prone crawl position, respectively.

**Figure 4:**
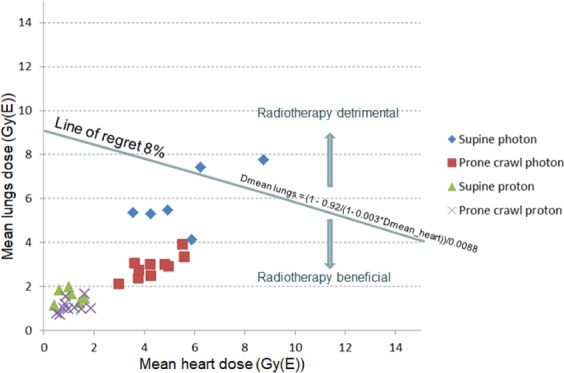
Risk-benefit classification of high-risk patients for cardiac events and lung cancer. Data points represent mean heart and mean lung doses of individual patients treated supine or prone crawl using photons or protons. Line of regret 8%: data points on the line represent 8% absolute 30-year survival loss from combined radiotherapy-related cardiac and lung cancer mortality.

Patients who are high-risk for lung cancer may simultaneously be high-risk for cardiac events by tobacco-related or other factors of cardiac disease. Figure 4, a plot of mean lung and heart doses for each patient shows that the combined mortality risk would outweigh a 30-year disease-specific survival benefit of 8% in 2/6 patients using supine photon plans but not using prone crawl photon or proton plans. Risk calculations based on cardiac and lung cancer mortality neglect risk-contributions from other radiation-induced cancers, such as esophageal, thyroid or contralateral breast cancer. Hence, radiation-related mortality risk is underestimated. Taylor’s data^11^ are based on a variety of prescription doses, the most common being 25*2.0 Gy. The prescription dose in this study was 15*2.67 Gy. A weakness of these risk calculations is that neither total dose nor fractionation could be taken into account.

Dose calculation algorithms might bias the comparison between photon and proton plans. Stray radiation was calculated in photon plans but not in proton plans. In proton PBS, stray radiation consists mainly of fast neutrons generated in the tissues of the patient and in the 4 cm thick range shifter of the nozzle used to generate superficial Bragg peaks. Integral dose from neutron radiation using proton PBS is much lower than stray radiation (mainly phantom scatter) using photons^34^. We observed good accuracy of Pinnacle convolution-superposition dose calculation algorithm in the build-up region for VMAT plans of whole brain radiotherapy^35^. We assumed that similar accuracy was obtained in VMAT for breast and lymph node radiotherapy.

Except for skin, the lowest doses to OAR are obtained by proton therapy in supine or prone crawl position. The drawbacks of proton therapy for adjuvant breast cancer treatment are increased skin toxicity, cost and availability. Using constraints to skin, PBS optimization could reduce dose in the most superficial 5 mm to 90–95% of the WBI prescription dose but cannot reach the level of under-dosage offered by megavoltage photon beams at the depth of the germinative layer of the skin epithelium which is 0.1–0.2 mm below the surface at the treated breast.

## Conclusion

Target coverage was better for proton than for photon plans and equally good in supine as in prone crawl position plans for each radiation modality. Mean doses to organs-at-risk are generally lower for prone crawl than for supine positions and for proton than for photon plans. Survival benefit/risk ratio <1 occurred for supine photon plans. Proton plans showed the lowest radiation-related mortality risks.

## Data Availability

The datasets generated during and/or analyzed during the current study are not publicly available due to patient privacy rights but are available in an anonymized form from the corresponding author on reasonable request.

## References

[1] Clarke M (2005). Effects of radiotherapy and of differences in the extent of surgery for early breast cancer on local recurrence and 15-year survival: An overview of the randomised trials. Lancet..

[2] Darby S (2011). Effect of radiotherapy after breast-conserving surgery on 10-year recurrence and 15-year breast cancer death: Meta-analysis of individual patient data for 10,801 women in 17 randomised trials. Lancet..

[3] Berrington de Gonzalez A (2010). Second solid cancers after radiotherapy for breast cancer in SEER cancer registries. Br J Cancer..

[4] Grantzau T, Thomsen MS, Vaeth M, Overgaard J (2014). Risk of second primary lung cancer in women after radiotherapy for breast cancer. Radiother Oncol..

[5] Darby SC (2013). Risk of ischemic heart disease in women after radiotherapy for breast cancer. N Engl J Med..

[6] Henson KE, McGale P, Taylor C, Darby SC (2013). Radiation-related mortality from heart disease and lung cancer more than 20 years after radiotherapy for breast cancer. Br J Cancer..

[7] Mulliez T (2013). Hypofractionated whole breast irradiation for patients with large breasts: A randomized trial comparing prone and supine positions. Radiother Oncol..

[8] Veldeman, L. *et al*. The 2-Year Cosmetic Outcome of a Randomized Trial Comparing Prone and Supine Whole-Breast Irradiation in Large-Breasted Women. *Int J Radiat Oncol Biol Phys*. Jul 15, **95**(4), 1210–7 (2016). 10.1016/j.ijrobp.2016.03.003 27209501

[9] Mulliez T (2015). Heart dose reduction by prone deep inspiration breath hold in left-sided breast irradiation. Radiother Oncol..

[10] Shin, S. M. *et al*. Breast, chest wall, and nodal irradiation with prone set-up: Results of a hypofractionated trial with a median follow-up of 35 months. *Pract Radiat Oncol*. **6**(4), e81–8, 10.1016/j.prro.2015.10.022 (2016 Jul-Aug). 10.1016/j.prro.2015.10.022 26723552

[11] Taylor, C. W. *et al* Exposure of the Heart in Breast Cancer Radiation Therapy: A Systematic Review of Heart Doses Published During 2003 to 2013. *Int J Radiat Oncol Biol Phys*. 15, **93**(4), 845–53 (2015). 10.1016/j.ijrobp.2015.07.2292 26530753

[12] Aznar, M. C., Duane, F. K., Darby, S. C., Wang, Z. & Taylor, C. W. Exposure of the lungs in breast cancer radiotherapy: A systematic review of lung doses published 2010–2015. *Radiother Oncol*. 2018, **126**(1), 148–154 (2018). 10.1016/j.radonc.2017.11.022 PMC5807032 29246585

[13] Taylor, C. *et al*. Early Breast Cancer Trialists’ Collaborative Group. Estimating the Risks of Breast Cancer Radiotherapy: Evidence From Modern Radiation Doses to the Lungs and Heart and From Previous Randomized Trials. *J Clin Oncol*. 20, **35**(15), 1641–1649 (2017). 10.1200/JCO.2016.72.0722 PMC5548226 28319436

[14] Thorsen, L. B. *et al*. DBCG-IMN: A Population-Based Cohort Study on the Effect of Internal Mammary Node Irradiation in Early Node-Positive Breast Cancer. *J Clin Oncol*. 1, **34**(4), 314–20 (2016). 10.1200/JCO.2015.63.6456 26598752

[15] Boute B (2017). . Potential benefits of crawl position for prone radiation therapy in breast cancer. J Appl Clin Med Phys..

[16] Deseyne P (2017). Whole breast and regional nodal irradiation in prone versus supine position in left sided breast cancer. Radiat Oncol..

[17] Verhoeven K (2015). Vessel based delineation guidelines for the elective lymph node regions in breast cancer radiation therapy - PROCAB guidelines. Radiother Oncol..

[18] Verhoeven K (2016). Vessel based delineation guidelines for the elective lymph node regions in breast cancer radiation therapy - PROCAB guidelines. Radiother Oncol..

[19] Offersen BV (2016). ESTRO consensus guideline on target volume delineation for elective radiation therapy of early stage breast cancer, version 1.1. Radiother Oncol..

[20] Feng M (2011). Development and validation of a heart atlas to study cardiac exposure to radiation following treatment for breast cancer. Int J Radiat Oncol Biol Phys..

[21] De Gersem W, Claus F, De Wagter C, Van Duyse B, De Neve W (2001). Leaf position optimization for step-and-shoot IMRT. Int J Radiat Oncol Biol Phys..

[22] Berwouts D (2016). Intensity modulated arc therapy implementation in a three phase adaptive (18)F-FDG-PET voxel intensity-based planning strategy for head-and-neck cancer. Radiat Oncol..

[23] Zenklusen SM, Pedroni E, Meer D, Bula C, Safai S (2011). Preliminary investigations for the option to use fast uniform scanning with compensators on a gantry designed for IMPT. Med Phys..

[24] Lomax AJ (2001). Intensity modulated proton therapy: a clinical example. Med Phys. Mar.

[25] Lomax AJ, Pedroni E, Rutz H, Goitein G (2004). The clinical potential of intensity modulated proton therapy. Z Med Phys..

[26] Early Breast Cancer Trialists’ Collaborative Group (2011). Effect of radiotherapy after breast-conserving surgery on 10-year recurrence and 15-year breast cancer death: Meta-analysis of individual patient data for 10,801 women in 17 randomised trials. Lancet..

[27] Early Breast Cancer Trialists’ Collaborative Group (2014). Effect of radiotherapy after mastectomy and axillary surgery on 10-year recurrence and 20-year breast cancer mortality: Meta-analysis of individual patient data for 8135 women in 22 randomised trials. Lancet..

[28] Hennequin, C. *et al*. Ten-year survival results of a randomized trial of irradiation of internal mammary nodes after mastectomy. *Int J Radiat Oncol Biol Phys*. 1. **86**(5), 860–6 (2013). 10.1016/j.ijrobp.2013.03.021 23664327

[29] Poortmans, P. M. *et al*. EORTC Radiation Oncology and Breast Cancer Groups. Internal Mammary and Medial Supraclavicular Irradiation in Breast Cancer. *N Engl J Med*. 23, **373**(4), 317–27 (2015). 10.1056/NEJMoa1415369 26200978

[30] Kainz K (2012). Simultaneous irradiation of the breast and regional lymph nodes in prone position using helical tomotherapy. Br J Radiol..

[31] Parkes MJ (2016). Safely prolonging single breath-holds to >5 min in patients with cancer; feasibility and applications for radiotherapy. Br J Radiol..

[32] Péguret N (2016). Apnea-like suppression of respiratory motion: First evaluation in radiotherapy. Radiother Oncol..

[33] Smolina K, Wright FL, Rayner M, Goldacre MJ (2012). Determinants of the decline in mortality from acute myocardial infarction in England between 2002 and 2010: linked national database study. BMJ..

[34] Hälg, R. A. *et al* Measurements of the neutron dose equivalent for various radiation qualities, treatment machines and delivery techniques in radiation therapy. *Phys Med Biol*. 21, **59**(10), 2457–68 (2014). 10.1088/0031-9155/59/10/2457 24778349

[35] De Puysseleyr A (2014). Hair-sparing whole brain radiotherapy with volumetric arc therapy in patients treated for brain metastases: dosimetric and clinical results of a phase II trial. Radiat Oncol..

